# Hippocampal Mitochondrial Transplantation Alleviates Age-Associated Cognitive Decline via Enhancing Wnt Signaling and Neurogenesis

**DOI:** 10.1155/2022/9325302

**Published:** 2022-05-31

**Authors:** Zhaoyichun Zhang, Di Wei, Zijie Li, Hanfeng Guo, Yin Wu, Jianying Feng

**Affiliations:** ^1^School of Stomatology, Zhejiang Chinese Medical University, Hangzhou 310053, China; ^2^Department of Pharmacy, Xi'an High-tech Hospital, Xi'an 710075, China; ^3^School of Basic Medicine, Air Force Military Medical University, Xi'an 710032, China

## Abstract

Gradual cognition decline and mitochondrial dysfunction are two notable changes closely associated with aging. Enhancing mitochondrial function has been assumed to be antiaging. However, most current mitochondria-promoting agents usually target 1-2 aspects of mitochondrial function. In the present study, we transplanted mitochondria isolated from young mice into the hippocampus of aged mice, which presumably boost mitochondrial function more thoroughly, examined the effects on cognition, and explored the possible underlying mechanism. Our data showed that exogenous mitochondria were efficiently internalized by nestin-positive neural progenitors in the hippocampus. Mitochondrial transplantation quickly increased ATP levels, enhanced the activity of mitochondrial complexes I, II, and IV, and decreased Tom20 expression in the hippocampus. In regard of cognitive function, mitochondria-treated mice displayed a remarkable improvement of novel object recognition and spatial memory. Utilizing the Wnt signaling reporting mouse line, TOPGAL mice, we detected activated canonical Wnt signaling in the neural progenitors of the mitochondria-treated hippocampus. Further, BrdU labeling showed that exogenous mitochondria significantly stimulated neural progenitor neurogenesis and proliferation. Taken together, our data demonstrated that exogenous mitochondria from young mice might be a novel way of rejuvenating the function of hippocampal neural progenitors to exert antiaging effects.

## 1. Introduction

Aging is a process of deterioration of physiological function of multiple organs [1], particularly brain function [2]. Gradual impairment of cognition and memory is the hallmark change of the aged brain [3]. As one of the key brain regions for memory and cognition, the hippocampus has been well studied for its involvement in aging and age-associated memory deficit [4, 5]. Improving the function of the hippocampus is supposed to be effective in antiaging.

At the cellular level, one key feature of aging is the dysfunction of mitochondria in multiple tissues. Mitochondria play various roles, including regulating metabolism, producing ATP, maintaining calcium homeostasis, regulating intracellular redox balance, and communicating with the nucleus [6–8]. In aged cells, most aspects of mitochondrial function are compromised, resulting in chronic oxidative stress and metabolic abnormality, which eventually lead to the phenotype of aging [9, 10]. In the hippocampus, a brain region of high energy consumption, mitochondria are more fragile for damage. Increasing evidence has demonstrated that hippocampal synaptic mitochondrial impairment is strongly associated with neuronal degeneration in Alzheimer's disease [11]. Many studies have attempted to enhance mitochondrial function for alleviating age-associated cognition deterioration. However, most of the current studies can only target one or two aspects of mitochondrial function [12]. Fully rejuvenating mitochondria in the aged hippocampus is still a challenging issue in the field.

Recently, several groups reported that grafting healthy mitochondria into the injured or ischemic cerebral cortex could restore mitochondrial function and dramatically reduce secondary injury [13, 14], which inspired the idea of transplanting “young” mitochondria into the aged hippocampus. In this research, our team explored the roles of transplanting mitochondria from young mice in the cognition of aged mice and explored the possible underlying mechanism.

## 2. Materials and Methods

### 2.1. Animals

TOPGAL mice were obtained from Jax Laboratory (stock number, 004623. https://www jax org/). Male C57 mice of 2 months (young) and 20 months (aged) were bought and housed in the animal facility of the Zhejiang Chinese Medical University (ZCMU). The animals could eat and drink freely. Every animal assay was completed as per the guideline for experimentation of animal welfare and utilization of ZCMU and complied with the ARRIVE guideline. Our animal assays were accepted by the Ethical Board of Animal Welfare and Utilization of ZCMU (animal ethical approval number: 2021–0109).

### 2.2. Mitochondria Separation, Quantification, and Transplantation

Mitochondria were separated from the liver of young mice (2-month-old mice). About 100 mg tissular sample was acquired via dissection and subjected to homogenization via mitochondrion separation liquor (BC3270, Solarbio). After centrifugation at 1000 g for 5 min, the supernate was moved into another tube and subjected to centrifugation at 3500 g for 10 min. The sediment was resuspended in 15% percoll before it was centrifugated at 21000 g for 8 min for two times. After removing the percoll liquor, mitochondria were precipitated and washed. Subsequently, mtDNA was separated as delineated in the past [15]. Mitochondrial DNA was quantified as described [14]. Bilateral hippocampus transplantation was performed with the following coordinates: anterior-posterior, −1.9 mm; mediolateral, −0.7 mm; dorsoventral, and −2.2 mm from bregma and dura. Approximately, 1.5–2 × 10^6^ mitochondria were injected for each site.

### 2.3. Mitochondrial Complex Activity and ATP Contents

Activities of mitochondrial complexes I–V and ATP contents were supervised via the test tools. In short, mitochondria acquired from about 100 mg tissular sample were broken by the supersonic method under 20% maximal output power. The procedure was completed repetitively for 20 times, and afterwards, the complex activities and ATP levels were measured by a spectrophotometer.

### 2.4. Western Blot (WB)

Hippocampus samples were discreetly collected via dissection and subjected to lysis in the RIPA buffering solution with proteinase suppressors mixture. Protein level was identified via the BCA test. Protein specimens were isolated via SDS-PAGE and moved onto PVDF films. Films were subjected to blockade in TBS involving 0.1% (v/v) Tween 20 and 5% (w/v) skimmed milk prior to cultivation with the first antibodies. Mouse anti-Tom20 was incubated with the membrane overnight at 4°C. After washes, films were cultivated with antimouse second antibodies conjugated with HRP (Cat. CW0102S, CWBIO Company). The visualization of bands was realized via the ECL tool (Thermo). Imaging results were studied via Image *J*.

### 2.5. Immune Histochemistry

Mice were killed and subjected to intracardial perfusion. The cerebral tissular sample was subjected to cryoprotection with 20–30% saccharose. Serial slices (14–20 *μ*m thick) were sliced, with entire slices mounted onto 8 slides. For immune staining, slices were subjected to blockade in 0.01 M PBS and 3% BSA for 60 min. The first antibodies were utilized, including mouse anti-NeuN, rat anti-BrdU, rabbit anti-DCX (1 : 400; ab108319, Abcam), rabbit anti-*β*-gal (1 : 300; 11132, MP), and goat anti-Nestin (1 : 300; sc-21247, Santa Cruz). After incubation, slices were cultivated with relevant second antibodies. The nucleus was subjected to counterstaining in DAPI (1 : 1000, Sigma). Pictures were captured via a confocal microscopic device Olympus FV3000.

### 2.6. Behavior Assays

*Morris Water Maze Assay. The* maze comprised a circular container (Φ 180 cm), with walls of 70 cm. To train the mice for the assay, they were placed in water stochastically in one quadrant facing the walls. The time spent for the animals to find the platform was documented as escape time (those which failed to locate the platform in 1 min were provided with guidance). The animals were subjected to training 4 times daily for 4 days. On the 5^th^ day, animals were allowed to explore the maze in a free manner. Their moving traces and escape behaviors were documented via the SMART 3.0 program.

New object recognition assay. On day 1, animals were placed in the open field (40 × 40 × 35 cm) and allowed to explore freely for 10 min. On day 2, animals were first placed in the identical region with 2 identical lego blocks and allowed to explore freely for 10 min. After 60 min, 1 block was substituted by a new one with a diverse shape, and animals were allowed to explore freely for 5 min. On day 3, animals were examined as they were tested on day 2, and the new block was substituted by another new block. The ratio of recognition time spent on the new block and the old block was computed.

### 2.7. Statistical Analysis

For mitochondrial function, immunohistochemistry and western blotting, every group was tested in triplicate. For behavioral analysis, every group contained ≥8 mice. Data were displayed as averages ± *S E*. Data meeting the normality and homoscedasticity were studied via Student's *t*-test or ANOVA. For the data failed to meet the criteria, the Mann–Whitney *U* test, Kruskal–Wallis H test, or Wilcoxon signed-rank test was completed via SPSS l6.0 (America). *P* < 0.05 had significance on statistics.

## 3. Results

### 3.1. Transplantation of Mitochondria from Young Mice Restores Mitochondrial Function in Aged Hippocampus

**Figure 1:**
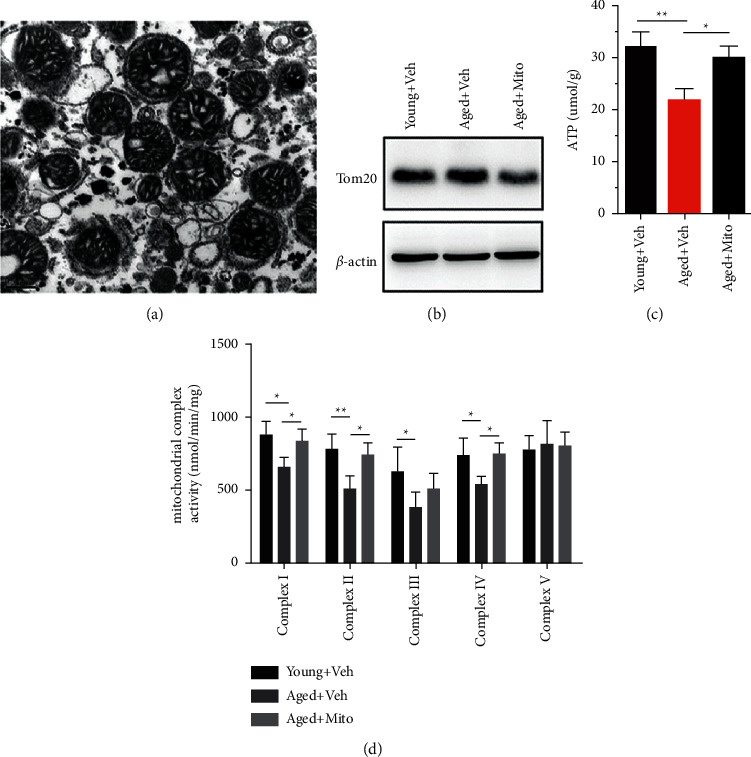
Restoration of mitochondrial function in the aged hippocampus by mitochondria transplantation. (a) Representative electron microscopic images of isolated mitochondria. (b) Western blots of Tom20 in young mice treated with vehicle, aged mice treated with vehicle, and aged mice treated with mitochondria. (c) ATP levels in the hippocampus of young mice injected with vehicle, aged mice injected with vehicle, and aged mice injected with mitochondria. (d) Mitochondrial complexes I–V activities of young mice treated with vehicle, aged mice treated with vehicle, and aged mice treated with mitochondria. The restoration of Tom20 expression, ATP level, and respiratory complex activity by exogenous mitochondria should be noted. Values represent the mean ± S.E.M. ^*∗*^(*P*) < 0.05. ^*∗*^(*P*) < 0.001. Veh, vehicle. Mito, mitochondria.

To test if mitochondria from young mice could improve the overall mitochondrial function in the hippocampus of aged mice, we isolated mitochondria from the liver of 2-month-old mice and immediately injected approximately 1.5–2 × 10^6^ fresh mitochondria into each hippocampus of 20-month-old mice. The purity and integrity of mitochondria were verified by the electron microscope (Figure 1(a)). At 3 days after graft, we dissected the hippocampus and examined the overall properties of mitochondrial function. The levels of Tom20 (a marker of mitochondrial damage) in the aged hippocampus treated with vehicle were higher than those in the young hippocampus but lower than those in the aged hippocampus treated with mitochondria (Figure 1(b)). The levels of ATP in the hippocampus of aged mice treated with vehicle were significantly lower than those in young mice but recovered in aged mice treated with mitochondria (Figure 1(c)). The activity of mitochondrial complexes I, II, III, and IV was significantly reduced in the hippocampal region of aged mice, in contrast to the hippocampus of young mice (Figure 1(d)). Following mitochondrial transplantation, the activity of mitochondrial complexes I, II, and IV was restored to levels similar to that in young mice (Figure 1(d)). These data indicated that mitochondrial transplantation could restore the overall mitochondrial function in the hippocampus of aged mice.

### 3.2. Hippocampal Transplantation of Mitochondria from Young Mice Alleviates Cognition Impairment in Aged Mice

**Figure 2:**
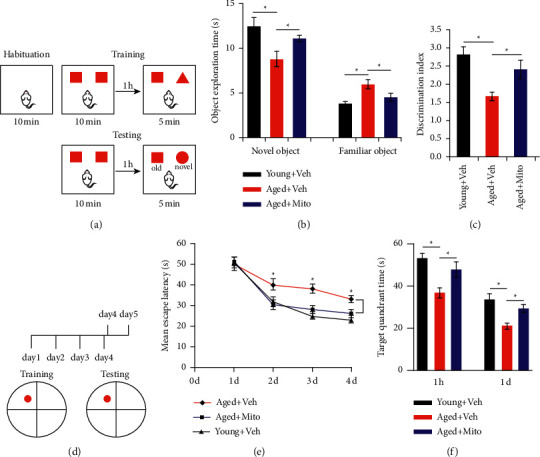
Effects of hippocampal mitochondria transplantation on the cognition of aged mice. (A–C) The novel object recognition test of young mice treated with vehicle, aged mice treated with vehicle, and aged mice treated with mitochondria. Notice the decline of novel object recognition in aged mice and improvement of novel object recognition in aged mice treated with mitochondria. (D–F) Morris water maze test of young mice injected with vehicle, aged mice injected with vehicle, and aged mice injected with mitochondria. The reduced escaping latency and prolonged target quadrant staying of aged mice treated with mitochondria should be noted. Values represent the mean ± S.E.M. ^*∗*^(*P*) < 0.05. Veh, vehicle. Mito, mitochondria.

In the new object assay evaluating the mice's recognition memory, aged mice (treated with vehicle) spent nearly 32% less time in terms of new object exploration over young adult mice (Figures 2(a) and 2(b)). Aged mice which received exogenous mitochondria showed significantly longer time in terms of new object exploration and a greater discrimination index (Figures 2(b) and 2(c)). In Morris water maze assay assessing space memory (Figure 2(d)), aged mice treated with mitochondria utilized remarkably less time in terms of escape from the training day 2 (Figure 2(e)), indicating the improvement of spatial memory. Moreover, aged mice treated with mitochondria spent remarkably longer time within the target quadrant at 60 min and 24 h posterior to 4-day training in contrast to their counterparts exposed to vehicle (Figure 2(f)). These data demonstrated that transplantation of mitochondria from young mice could effectively improve cognition function in aged mice.

### 3.3. Exogenous Mitochondria Activate Wnt Signaling and Enhance Neurogenesis in Hippocampus

**Figure 3:**
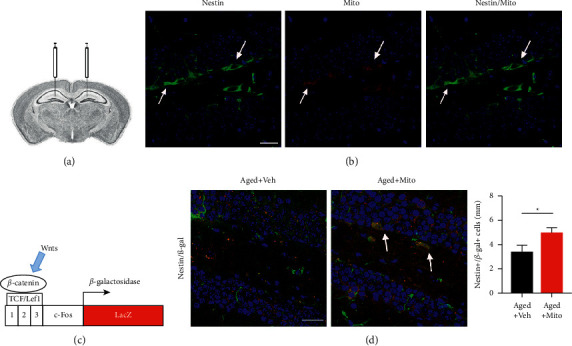
Internalization of exogenous mitochondria and activation of Wnt signaling in the hippocampus. (A, B) Injection of Mito-tracker-labeled mitochondria and immunostaining of nestin at 24 h after transplantation. Mito-tracker-labeled cells are mainly nestin-positive cells. (C, D) Transgenic strategy of TOPGAL mice and double-immunostaining of nestin/*β*-gal in aged mice treated with vehicle or mitochondria. There were significantly more nestin/*β*-gal levels in mice following mitochondrial transplantation. Values represent the mean ± S.E.M. ^*∗*^*P* < 0.05. Veh, vehicle. Mito, mitochondria. Arrows point to double-stained cells. Bars = 50 *μ*m.

To explore the possible underlying mechanisms, we examined which cells internalized the exogenous mitochondria in the hippocampus. Before transplantation, mitochondria were labeled by the/a Mito-tracker. Mito-tracker-labeled cells were analyzed by immunohistochemistry at 24 h posttransplantation (Figure 3(a)). The results showed that most of the Mito-tracker-labeled cells localized horizontally at the basal layers of the dentate gyrus. Double-immunostaining revealed that these Mito-tracker-positive cells coexpress well with nestin (Figure 3(b)), suggesting the neural progenitor identity of these cells. There are two populations of progenitors in the hippocampus, active neural stem cells (aNSCs), and quiescent neural stem cells (qNSCs) [16], which could be roughly identified by morphology and play important roles in the cognitive function of the hippocampus [17], namely, aNSCs are horizontal and qNSCs are radial [16]. To explore if exogenous mitochondria regulate the behavior of neural progenitors in the hippocampus, we focused on Wnt/*β*-catenin signal transduction, a pivotal signal path, which regulates the balance of qNSCs and aNSCs [16], via a Wnt signal transduction reporting mice line, TOPGAL mice [18]. In these mice, the expression of *β*-gal was controlled by the c-fos promoter which had 3 TCF/LEF1 binding sites at its 5′ end (Figure 3(c)). At 5 days following mitochondria transplantation, a significant increase of *β*-gal/nestin-positive cells was observed in aged mice (Figure 3(d)), indicating that Wnt signaling was activated or enhanced by mitochondrial transplantation.

**Figure 4:**
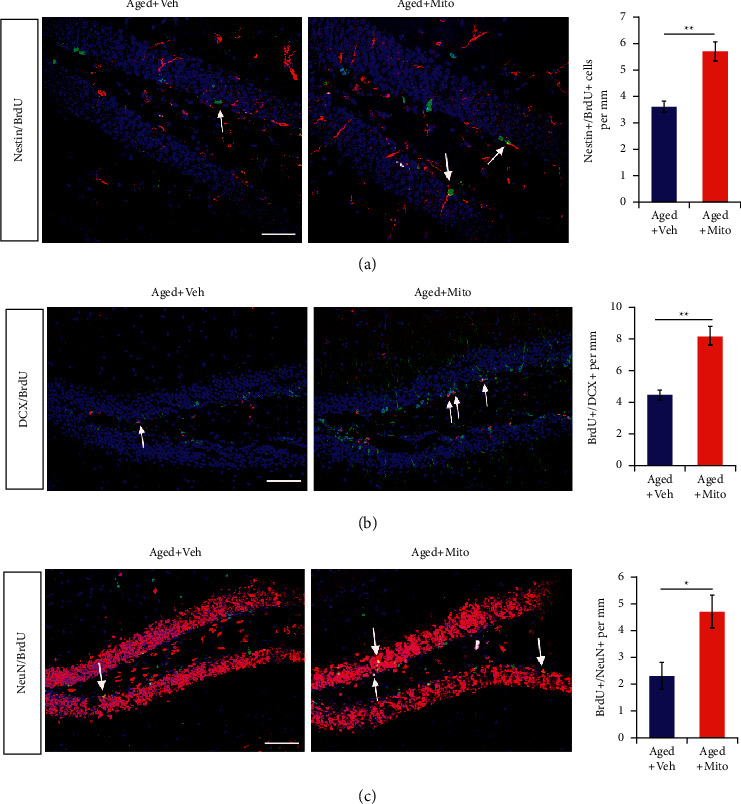
Effects of exogenous mitochondria in hippocampal neurogenesis. (a) Immunohistochemistry of nestin/BrdU in aged mice treated with vehicle or mitochondria at 2 h after last BrdU injection. (b) Immunohistochemistry of DCX/BrdU in aged mice treated with vehicle or mitochondria at 3 d after last BrdU injection. (c) Immunohistochemistry of DCX/BrdU in aged mice treated with vehicle or mitochondria at 14 d after last BrdU injection. Values represent the mean ± S.E.M. ^*∗*^*p* < 0.05. ^*∗∗*^*p* < 0.01. Veh, vehicle. Mito, mitochondria. Arrows point to double-stained cells. Bars = 100 *μ*m.

As Wnt signaling stimulates the proliferation of hippocampal neural progenitors and neurogenesis [19], we further evaluated if exogenous mitochondria affected the proliferation of neural progenitors and corresponding neurogenesis. BrdU was administered 1 time daily for 3 consecutive days. Mice were killed at 2 h, 3 d, and 14 d following last BrdU administration to assess the proliferation of neural progenitors and generation of immature neurons and mature neurons, respectively. The results showed that, in aged mice treated with exogenous mitochondria, there were significantly more nestin/BrdU-, DCX/BrdU-, and NeuN/BrdU-positive cells in the dentate gyrus of the hippocampal region, in contrast to the controls exposed to vehicle (Figures 4(a)–4(c)). Above results suggested that exogenous mitochondria from young mice activated Wnt signaling and enhanced neurogenesis in the hippocampus.

## 4. Discussion

In the present study, we tested the hypothesis that mitochondria isolated from young mice could restore the overall mitochondrial function in the hippocampus and improve cognition in aged mice. By injecting freshly prepared mitochondria from 2-month-old mice into the hippocampus of 20-month-old mice, we observed enhanced novel object recognition and spatial memory in mitochondria-treated aged mice. Interestingly, these exogenous mitochondria were efficiently internalized by hippocampal neural progenitors. In addition, transplantation of mitochondria led to activation of canonical Wnt signal transduction and increased neurogenesis in the hippocampus of aged mice, indicating that mitochondria from young mice might be able to rejuvenate the function of hippocampal neural progenitors to exert antiaging effects.

Previous studies have demonstrated that mitochondrial dysfunction in the hippocampus is an important cellular change closely associated with aging [20]. The results herein revealed that the reduction of mitochondrial respiratory complex activity in the aged hippocampus was inconsistent with a previous report [21]. Recent 3D electron microscopic study demonstrated that mitochondrial morphology in different compartments of CA-1 and dentate gyrus neurons changed with aging [22]. Stimulating mitochondrial genesis and enhancing complex activity have been thought as efficient ways of developing antiaging medicine. However, researchers could only improve certain aspects of mitochondrial function. So far as we know, only one paper reported mitochondria transplantation, which presumably could restore mitochondrial function more thoroughly and improved cognition function in severe diabetic mice [23]. In that study, exogenous mitochondria were injected into the lateral ventricle [23], which was not optional as the size of mitochondria is too large to be transferred through extracellular diffusion and might affect multiple brain areas. In the present study, we injected mitochondria locally into the hippocampus and observed notable enhancement of cognition, indicating that injecting exogenous healthy mitochondria may be a new promising strategy for rejuvenating hippocampal function in aged mice.

More importantly, our data showed that exogenous mitochondria were internalized by horizontal neural progenitors and stimulated neurogenesis in the hippocampus. During aging, neural progenitor proliferation and neurogenesis in the hippocampus were gradually downregulated, which was thought as one important cause of cognition decline [24, 25]. Therefore, enhancement of neurogenesis by exogenous mitochondria may partially explain the improvement of memory. The possibility that neurons uptake exogenous mitochondria is not excluded, which may also contribute to cognition improvement. As water Morris maze assay revealed better performance of mitochondria-treated mice at 1 month after mitochondrial transplantation, which was longer than the life span of mitochondria in neurons, the long-term cognition improvement may be the results of enhanced neurogenesis.

The activation of Wnt signaling following mitochondria transplantation is interesting. Previous studies have demonstrated that Wnt signaling could regulate mitochondrial function [26, 27]. In the hippocampus, Wnt signaling is high during development and fades off in adults. Our observation indicated that “young” or healthy mitochondria may help to maintain a high level of intracellular Wnt signaling. How mitochondria regulates Wnt signaling is worthy to be further investigated.

## Data Availability

The datasets used and analyzed during the current study are available from the corresponding author on reasonable request.
